# Comment on “Controversies about Interspinous Process Devices in the Treatment of Degenerative Lumbar Spine Diseases: Past, Present, and Future”

**DOI:** 10.1155/2017/6545361

**Published:** 2017-05-11

**Authors:** Alessandro Landi, Fabrizio Gregori, Giovanni Grasso, Cristina Mancarella, Roberto Delfini

**Affiliations:** ^1^Department of Neurology and Psychiatry, Division of Neurosurgery A, Sapienza University of Rome, Rome, Italy; ^2^Department of Biomedicine and Clinical Neurosciences, Neurosurgical Clinic, University of Palermo, Palermo, Italy

We read with extreme interest the article by Gazzeri et al., entitled “Controversies about Interspinous Process Devices in the Treatment of Degenerative Lumbar Spine Diseases: Past, Present, and Future” [1], published in Biomed Research International Volume 2014, Article ID 975052. In this manuscript the authors present a literature review about commercialized interspinous devices, with particular attention to their possible clinical indications. The authors describe some of those devices, defining their biomechanical properties and the therapeutic capabilities, analysing the therapeutic results available in the literature for some devices. Conclusion of the article is that the available scientific evidence is not sufficient to permit conclusions for or against the placement of an interspinous spacer for the treatment of lumbar stenosis (2011 NASS guidelines). The authors also add that if related to their minimal invasiveness, interspinous spacers seem to have a robust pathophysiological substrate and promise to play an important role in the future degenerative lumbar microsurgery, especially in the older population. The article is really interesting but deserves an in-depth comment on many topics. Firstly it is mandatory to focus on the pathological substrate responsible for the disease that those devices are going to treat.

Lumbar stenosis, identified by the authors as the primary pathological condition to be treated with IPD, is described, from a pathophysiological point of view, as the last stage of the degenerative cascade, the process of degeneration of the motor spinal unit described by Kirkaldy-Willis that identifies the origin of the degeneration in the segmental hypermobility [2].

The grade of instability varies according to the grade of hypermobility, increasing from a first-phase (modest disc and articular damage) defined microinstability, to a second phase of clear instability, until a final phase of stenoinstability. It is important to remember that those three phases evolve in a progressive manner, in relation to the progressive increase of the instability and to the increase of the osteoligamentous hypertrophic reaction to the hypermobility.

Therefore, the stenosis arises between the second and the third phase of the degenerative cascade and is the reaction of the motor spinal unit to instability. The concept of instability is the basis of the whole degenerative process upon which are based the observations regarding IPDs.

From these considerations the surgery of dynamic stabilization, or motion preservation surgery, has evolved into “dynamic neutralization of the instability,” based upon the concept that aim of a dynamic device is not the motion of the motor unit, but the neutralization of the excessive degrees of mobility responsible for the degenerative disease.

Those devices have been conceived and developed as dynamic stabilization devices and motion preserving devices, capable of maintaining the mobility of the motor unit. Therefore, the treatment of the stenosis, both hard and soft stenosis, has to be strongly related to instability as basic pathological condition. 

*Pathophysiology and Mechanism of Action*. IPDs, as the authors describe, have been conceived as interspinous distraction devices, aiming at the distraction of the posterior part of the spinal unit, reducing the stenosis thanks to the stretch of the ligamentum flavum and the enlargement of the neural foramina. Considering this mechanism of action, it is important to do some considerations.

- IPDs, as described by the authors, have as their primary indication the stenosis associated with moderate neurogenic claudication and/or radiculopathy regressing with the anterior flexion of the trunk. They assess that the action of IPDs aims to stretch the ligamentum flavum that, for the collapse of the disc, becomes redundant, occupying more space in the spinal canal. This is only partly true. It has been demonstrated and described in literature that ligamentum flavum, in case of stenosis, has an important hypertrophic component whose development is related to the attempt of the body to stretch the ligamentum itself. The hypertrophy is related to the variation of the ratio between elastic fibres and collagen fibres in the most superficial layer of the ligamentum flavum, due to the micro traumatism produced by metameric instability on the ligamentum. The inversion of the ratio causes a loss of elasticity and augments the rigidity of the ligament, increasing its volume [3–8]. It is clear how an IPD cannot have an effect on soft central stenosis, because its action is not effective on ligamentous hypertrophy, with scarce results on neurogenic claudication.
- The foraminal stenosis upon which the IPDs are intended to perform their action is constituted not only by the hypertrophy of the ligamentum flavum, but also by the hypertrophy of the articular masses and of the bony structures, configuring a hard and soft stenosis. In this situation, a great component of the painful and dysfunctional symptomatology is related to the stenosis of the lateral recess, sustained by the hypertrophic articular masses. This stenosis can be responsible for a reduction of the spinal canal dimensions of more than 80% and is responsible for both neurogenic claudication and radiculopathy. The recess stenosis does not involve the nerve root exiting through the foramen at the same level of the stenosis but involves the passing root emerging at the level below. For example, if the level involved by the stenosis is L4-L5, a foraminal stenosis involves the emerging root, in this case L4. A central and recess stenosis involves the root passing in the recess and emerging from the foramen located below, in this case L5. This kind of stenosis requires surgical treatment with decompression both of the canal and of the recess.
- The authors report that the use of IPDs has become an acceptable alternative for the treatment of lumbar instability. In light of the biomechanical considerations furnished by Kirkaldy-Willis about the pathological substrate responsible for the degenerative disease, it is extremely clear how an IPD can only and unavoidably accelerate the degenerative cascade if it is implanted on a patient with lumbar instability.
- IPD is responsible for a posterior distraction of the spinous processes. The direction of the distraction is not parallel to the vertebral endplates, so that the intradiscal pressure is not reduced in a homogeneous fashion. The posterior unload of the disc generates an overload on its anterior portion [9], forcing the dislocation of the nucleus pulposus posteriorly (the same mechanism is reproduced during the anterior flexion of the trunk, majorly responsible for the herniation of the nucleus pulposus) theoretically increasing the risk of disc herniation.
- On this purpose, there is an article [10] that analyses the various stabilization systems, both rigid and dynamic. From its results emerges the fact that only rigid stabilization with screws and rods in distraction can significantly reduce intradiscal pressure. Another study [9] underlines that the distraction resulting by the placement of an IPD reduces the load of the posterior annulus in extension, neutral, and flexion position, but the load of the anterior annulus is increased about 400% due to distraction. This demonstrates that IPDs increase the risk of progression of disc degeneration.
- The implant of an IPD, whether restricted or not, causes an anterior overload in an already degenerated disc, accelerating the existing process and causing an acceleration of the instability [11].
- The implantation of an IPD causes consequences on the entire spine; the distraction causes a reduction in lordosis of both the disc and the lumbar segment, with alterations in sagittal balance and spinopelvic alignment.
- The combined action of IPD and surgical decompression associated with interbody fusion is not clear. How an interbody fusion device with TLIF technique can be associated with an IPD? How can a rigid system and a dynamic system coexist in the same segment? Maybe the authors in point 2.6 refer to IFD, Interspinous Fusion Devices, whose biomechanical action is different from the one of IPD: in fact they are projected with the aim of bony fusion, not of motion preservation. Therefore, it is an important distinction about the classification of interspinous devices.

*IPD Classification*. The authors, in their review, make a classification of IPDs identifying static, dynamic, and fusion devices. This aspect needs to be clarified. Those devices are classified on the basis of their biomechanical mechanism in restricted, not restricted, and fusion devices (and not as specified by the authors in static and dynamic) [12]. Restricted IPDs are anchored to the above and below spinous processes, limiting the hypermovement during flexion and extension movements. Nonrestricted IPDs are not ligated to the interspinous processes, so that they have a control on hypermovement only during extension. Fusion devices have a completely different mechanism, because their aim is bony fusion of the spinal segment, and not its movement. For this reason, a distinction is needed between IPD (restricted and not restricted) and IFD (fusion devices), because they belong to two different categories, given that they have a completely different purpose [12].

*Cost-Effectiveness*. The cost analysis made by the authors about healthcare expense of IPD surgery versus decompression is shareable, but it is appropriate to mention that the high reintervention rate for IPD mobilization, progression of disc degeneration, and recurrence of pain increases the costs for the treatment of the pathology. Furthermore, decompressive surgery is less expensive if compared to the cost of the device alone. Moojen et al., in a 2013 article [13], showed that IPDs, in a controlled trial, had a reintervention rate of 29–38%, with an increase of the healthcare expense. In 2014 the SIPS group published a study [14] that analysed the cost-utility of IPDs: their results showed that “the implantation of IPDs as indirect decompressing device, leads to higher healthcare costs and do not improve quality of life after treatment compared with standard bony decompression. Therefore, implantation of IPD as indirect decompressing device is highly unlikely to be cost effective compared with bony decompression.”

*Indications*. Clinical indications about the use of IPDs have been furnished by the companies and during the years have been extended to many pathological conditions such as lumbar disc herniation [15] and lumbar instability [16]. Such indications have been more and more limited during the last years. For example, in foraminal stenosis, the indication has lost validity, given the acceleration of the segmental degenerative cascade caused by IPDs [17]. For central stenosis the gold standard treatment is the central decompression with laminectomy [14]. In spondylolisthesis IPDs increase the shear stress on the disc and the consequent slippage [18]. There is no indication for IPDs placement in either microinstability, black disc, or recurrent disc herniation [19]. Regarding this, in the last 24 months, many important evolutions have been highlighted on a scientific and legislative basis, both in Italy and the USA, aiming to regulate the unlimited use of those devices.

*Recent Evolutions*. Many papers in the last years [14, 17, 18, 20–22] have focused their attention on the high complications rate, high revision surgery rate, and high cost (if compared to decompressive surgery) of the IPDs. The North American Spine Society (NASS) has published in 2013 a revision [23] of the “evidence based clinical guideline for the diagnosis and treatment of degenerative lumbar spinal stenosis,” originally published in 2006. This revision makes a clinical analysis and the analysis of the available data about the treatment options for lumbar stenosis, assigning a grade of recommendation going from A to I, A being the absolute grade of recommendation (the gold standard), and I the lack of recommendation. IPDs have been classified as I: “there is insufficient evidence at this time to make a recommendation for the placement of an interspinous process spacing device in patient with lumbar spinal stenosis.” In Italy in 2013, during the same period of the NASS revision, a commission has been identified, composed of 2 neurosurgeons and 2 orthopaedic surgeons, aiming to analyse the clinical indications of IPDs and provide a critical analysis of the evidences available in literature, in the attempt to formulate a guideline. On the basis of this study, on January 23, 2015, a ministerial directive from the ministry of health has been published, subscribed by the SiNch, Italian Society of Neurosurgery, and SIOT, Italian Society of Orthopaedics and Traumatology, that clarifies many aspects about the use of those devices in our country. This publication suggests the main contraindications to the use of IPDs: lumbar instability, lumbar disc herniation, recurrent lumbar disc herniation, synovial cysts, hard stenosis, and osteoporosis, limiting the use of IPDs to patients with soft stenosis and high ASA score. Furthermore, this document dictates that the implant of an IPD has to be in the setting of controlled clinical trials, after the consent expressed by competent ethical committees and notified to the general direction of the medical devices. With this directive, the Italian Ministry of Health confirms the international guidelines published by NASS, in line with the global trend of reduction of IPDs use. 

*Conclusions*. The preservation of the physiological characteristics of the spine, aim of motion preservation surgery, should particularly be aimed towards the whole motor unit intended to be responsible for the segmental movement. In light of this, IPDs do not seem to respect the biomechanical characteristics of the motor unit, accelerating the degenerative process and worsening the clinical symptoms of patients. So this kind of device does not seem to have a definite and correct clinical indication at the moment. Despite this, IFDs with their main aim being the treatment of instability have a restricted range of clinical indications and their use can definitely be a resource for both the patient and the surgeon. In conclusion these implants must not become a trend but only a tool in the surgeon's hands. As every tool in surgery, the use of IPDs has to be based on a deep knowledge of their biomechanical consequences and of the potential complications of their action. An ideal management would consider a collaboration between surgeons, specialty societies, and governmental agencies, to better define Evidence Based Medicine-based guidelines and to verify the adherence to those guidelines, for a better patient care.
